# Sprouting and intussusceptive angiogenesis in postpneumonectomy lung growth: mechanisms of alveolar neovascularization

**DOI:** 10.1007/s10456-013-9399-9

**Published:** 2013-10-23

**Authors:** Maximilian Ackermann, Jan P. Houdek, Barry C. Gibney, Alexandra Ysasi, Willi Wagner, Janeil Belle, Johannes C. Schittny, Frieder Enzmann, Akira Tsuda, Steven J. Mentzer, Moritz A. Konerding

**Affiliations:** 1 Institute of Functional and Clinical Anatomy, University Medical Center of the Johannes Gutenberg-University Mainz, 55128 Mainz, Germany; 2 Laboratory of Adaptive and Regenerative Biology, Brigham and Women’s Hospital, Harvard Medical School, Boston, MA USA; 3 Institute of Anatomy, University of Bern, Bern, Switzerland; 4 Computer Tomography Lab, Institute of Geosciences, Johannes Gutenberg-University Mainz, Mainz, Germany; 5 Molecular and Integrative Physiological Sciences, Harvard School of Public Health, Boston, MA USA

**Keywords:** Intussusceptive angiogenesis, Pneumonectomy, Septal alveolarization, Corrosion cast, Synchrotron radiation tomographic microscopy, Lung surgery

## Abstract

In most rodents and some other mammals, the removal of one lung results in compensatory growth associated with dramatic angiogenesis and complete restoration of lung capacity. One pivotal mechanism in neoalveolarization is neovascularization, because without angiogenesis new alveoli can not be formed. The aim of this study is to image and analyze three-dimensionally the different patterns of neovascularization seen following pneumonectomy in mice on a sub-micron-scale. C57/BL6 mice underwent a left-sided pneumonectomy. Lungs were harvested at various timepoints after pneumonectomy. Volume analysis by microCT revealed a striking increase of 143 percent in the cardiac lobe 14 days after pneumonectomy. Analysis of microvascular corrosion casting demonstrated spatially heterogenous vascular densitities which were in line with the perivascular and subpleural compensatory growth pattern observed in anti-PCNA-stained lung sections. Within these regions an expansion of the vascular plexus with increased pillar formations and sprouting angiogenesis, originating both from pre-existing bronchial and pulmonary vessels was observed. Also, type II pneumocytes and alveolar macrophages were seen to participate actively in alveolar neo-angiogenesis after pneumonectomy. 3D-visualizations obtained by high-resolution synchrotron radiation X-ray tomographic microscopy showed the appearance of double-layered vessels and bud-like alveolar baskets as have already been described in normal lung development. Scanning electron microscopy data of microvascular architecture also revealed a replication of perialveolar vessel networks through septum formation as already seen in developmental alveolarization. In addition, the appearance of pillar formations and duplications on alveolar entrance ring vessels in mature alveoli are indicative of vascular remodeling. These findings indicate that sprouting and intussusceptive angiogenesis are pivotal mechanisms in adult lung alveolarization after pneumonectomy. Various forms of developmental neoalveolarization may also be considered to contribute in compensatory lung regeneration.

**Electronic supplementary material:**

The online version of this article (doi:10.1007/s10456-013-9399-9) contains supplementary material, which is available to authorized users.

## Introduction

In 2010, more than 50 million individuals worldwide were suffering from life-threatening end-stage lung diseases in the US alone, with 240.000 patients undergoing lung surgery [1, 2]. In contrast to humans, pneumonectomy in small laboratory animals results in compensatory lung growth with complete restoration of the lung capacity [3]. In humans, recent evidence suggests that compensatory growth may occur—but the time course is months to years rather than days to weeks [4]. One key element of this regenerative process is lung angiogenesis during the formation of new alveoli. New blood vessel formation after pneumonectomy shows many parallels to angiogenesis during normal lung development [5]. Between birth and adolescence in lung development, a 23-fold increase in the lung volume becomes apparent. Lung microvasculature grows even more: capillary volume increases by a factor of 35. The development of the alveolar capillary meshwork is a complex morphogenetic process requiring not only blood vessel and alveolus construction, but also the efficient matching of ventilation and blood flow.

In a previous paper on post-pneumonectomy lung growth we focused on the time course and distribution of proliferation and gene transcription [5]. These studies revealed heterogeneously distributed cell proliferation and gene transcription in the various lung lobes which peaked 6 days after pneumonectomy [5]. The highest activities were seen in the cardiac lobe of the right lung which was shifted into the empty left pleural cavity and the subpleural regions. We concluded that the heterogeneities were most likely attributable to the differences in local mechanical stretch load. Stretch appears to play a central role in the growth and development of many organs, particularly in the lung. Moreover, micromechanical stretch, shear-stress and changes in blood flow are assumed to be decisive factors in pulmonary angiogenesis besides the roles of various pro-angiogenic growth factors (e.g., VEGF, FGF, and PDGF) [6, 7].

Blood vessel growth can take place either by sprouting or by non-sprouting, intussusceptive angiogenesis. The latter is a well-characterized morphogenetic process which can be observed during growth and remodeling of pre-existing networks [8, 9]. A distinguishing anatomic feature of intussusceptive angiogenesis is the intussusceptive pillar. The intussusceptive pillar is a 1–5 μm [10] transvascular tissue bridge that spans the vessel lumen; its small size typically requires corrosion casting and scanning electron microscopy (SEM) or synchrotron radiation-based tomographic microscopy (SRXTM) for visualization. Physical expansion or growth of the pillar along the vessel axis between two branching points divides the lumen, resulting in vascular duplication. In contrast to sprouting angiogenesis, intussusceptive angiogenesis is a rapid recovery adaption of existing microvascular network that does not rely solely on immediate endothelial cell proliferation, but instead emerges due to a reorganization of endothelial cells and the invasion of endothelial precursor cells. In a previous paper [5] we have already provided evidence of the involvement of both sprouting and intussusceptive angiogenesis during post-pneumonectomy lung growth [5].

In the present report, we have investigated the different mechanisms of angiogenesis originating from pre-existing bronchial and pulmonary vessels in compensatory neoalveolarization after pneumonectomy in mice on a submicron-scale.

## Methods

### Animals

A total of 72 C57/B6 mice (Charles River Laboratories, Sulzfeld, Germany), 8–12 weeks old, housed in an approved animal care facility with 12-h light/dark cycles were used in the experiments. Food and water were provided ad libitum. The care of the animals was consistent with legal guidelines and was approved by the Institutional Animal Care and Use Committee of Rhineland-Palatinate (Koblenz, Germany).

### Pneumonectomy

Animals were anesthetized with an intraperitoneal injection of ketamine 100 mg/kg (Pfizer, Berlin, Germany) and Xylazine 6 mg/kg (Bayer, Leverkusen, Germany). The glottis was directly visualized and intubation carried out with a 20G catheter (B. Braun, Melsungen, Germany) connected to a Harvard rodent ventilator (Harvard Apparatus, Holliston, MA, USA) at 200 beats per min, 10 ml/kg, and a positive end-expiratory pressure of 2 cm H_2_O with a pressure-limited constant flow profile of 30 cm H_2_O. The pneumonectomy was performed through a left 5th intercostal space thoracotomy. With minimal manipulation of the lung, the hilum was ligated en bloc with a 5-0 surgical silk tie (Ethicon, Norderstedt, Germany). The entire left lung distal to the hilar ligature was excised, the lung removed, and the thoracotomy closed with interrupted 5-0 silk sutures (Ethicon). Once spontaneous muscle activity returned, the animal was extubated and transferred to a warming cage. Sham pneumonectomy involved an identical left thoracotomy incision and closure without surgical manipulation of the left lung. Lungs were harvested 0, 3, 5, 7, 9, 14, and 21 days after pneumonectomy.

### Scanning electron microscopy

After systemic heparinization with 2,000 U/kg heparin IP, the mice were thoracotomized in deep anesthesia. The pulmonary artery was cannulated through the right ventricle with an olive-tipped cannula and perfused with 5 ml of 37 °C saline, followed by 5 ml of a buffered 2.5 % glutaraldehyde solution (Sigma Aldrich, Munich, Germany) at pH 7.40. After casting of the microcirculation with 3 ml of the polyurethane-based casting resin PU4ii (vasQtec, Zurich, Switzerland) and caustic digestion, the microvascular corrosion casts were imaged after coating with gold in an argon atmosphere with a Philips ESEM XL30 scanning electron microscope (Philips, Eindhoven, Netherlands). Stereopair images were obtained by using tilt angles of 6°.

### MicroCT

CT scans alone were obtained with a GE eXplore 120 CT scanner at 50 μm/pixel resolution. The serial DICOM images were exported for use in custom-made finite element software, as previously described [11]. 3D finite element geometric models of the murine lung pre- and post-pneumonectomy underwent volume analysis aspreviously described by Filipovic et. al [12].

### Synchrotron radiation tomographic microscopy

The samples were scanned at an X-ray wavelength of 1 Å (corresponding to an energy of 12.398 keV) at the microtomography station of the Materials Science Beamline at the Swiss Light Source of the Paul Scherrer Institut (Villigen, Switzerland). The monochromatic X-ray beam (ΔE/E = 0.014 %) was tailored by a slits system to a profile of 1.4^2^ mm^2^. After penetration of the sample, X-rays were converted into visible light by a thin Ce-doped YAG scintillator screen (Crismatec Saint-Gobain, Nemours, France). Projection images were further magnified by diffraction-limited microscope optics and finally digitalized by a high-resolution CCD camera (Photonic Science, East Sussex, United Kingdom). For the tissue samples, the optical magnification was set to ×10, vascular casts were scanned without binning with an optical magnification, resulting in a voxel size 0.7^3^ μm^3^. For each measurement, 1,001 projections were acquired along with dark and periodic flat field images at an integration time of 4 s each without binning. Data were postprocessed and rearranged into flat field-corrected sinograms online. Reconstruction of the volume of interest was performed on a 16-node Linux PC Farm (Pentium 4, 2.8 GHz, 512 megabytes RAM) using highly optimized filtered back-projection. A global thresholding approach was used for surface rendering. For 3-D visualization and surface rendering, Amira software (Burlington, MA, USA) was installed on an Athlon 64 3500-based computer.

### Light and transmission electron microscopy

Lungs designated for microscopy were harvested after cannulation of the trachea. The tissue was fixed by instillation of 2.5 % buffered glutaraldehyde into the bronchial system followed by the instillation of 50 % Tissue-Tek^®^ O.C.T.™ (Fisher Scientific, Schwerte, Germany) in saline. Post-fixation samples of the cardiac lobe were harvested and processed according to standard protocols and embedded in Epon (Serva, Heidelberg, Germany). Semi-thin sections (0.5 μm) were stained with tolouidine blue (Sigma Aldrich, Munich, Germany) and analysed with a Zeiss Axiophot microscope (Zeiss, Oberkochen, Germany). 700 Å ultrathin sections were analysed using a Leo 906 digital transmission electron microscope (Leo, Oberkochen, Germany).

## Results

### Compensatory lung growth in the cardiac lobe

**Fig. 1:**
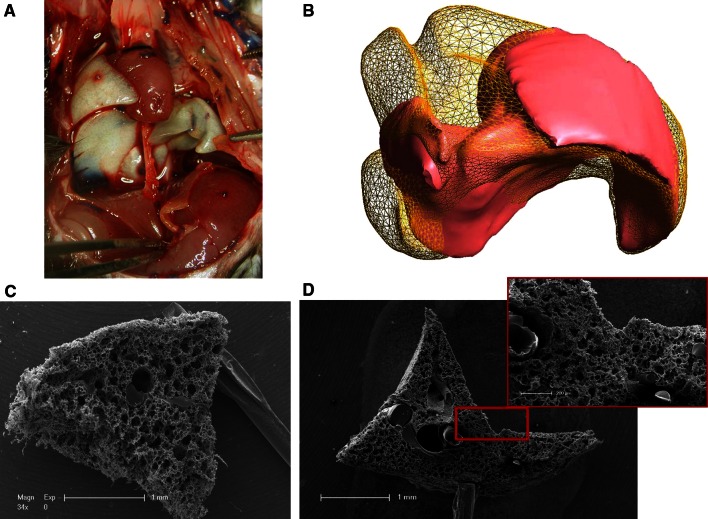
Compensatory lung growth in the cardiac lobe. Left pneumonectomy in mice results in compensatory growth of the right lung. The cardiac lobe in particular extends dramatically into the left pleural cavity enabling restoration of the vital capacity within 21 days. **a** The cardiac lobe fills out the left pleural cavity almost completely, already 14 days after surgery. **b** Finite element reconstructions of the cardiac lobe originating from microCT data reveal an increase of 143 percent in volume 14 days after pneumonectomy. Reconstruction created by Nenad Filipovic, University of Kragujevac, Serbia. **c**, **d** Contrary to the vasculature of control cardiac lobes (**c**), low power SEM images of microvascular corrosion casts showing heterogeneous vascular densities and distributions 7 days after surgery

The removal of the left lung results in a compensatory growth of the right lung with the highest activity in the cardiac lobe that expands dramatically in the left pleural chest (Fig. 1a). MicroCT measurements of different volumes of thr cardiac lobe after pneumonectomy showed a steep augmentation of 143 % on day 14 as a result of the high increase in proliferation that peaks on day six after pneumonectomy as detailed in our previous work. Spatial visualization of the increase in cardiac lobe by finite element reconstructions showed a volume uptake from the regions close to the heart towards the chest wall (Fig. 1b). SEM evaluation of microvascular corrosion casts demonstrated a heterogenous distribution of vascular growth spots which were highly evident in the indentation close to the heart (Fig. 1c, d).

### Spatial growth heterogeneity

**Fig. 2:**
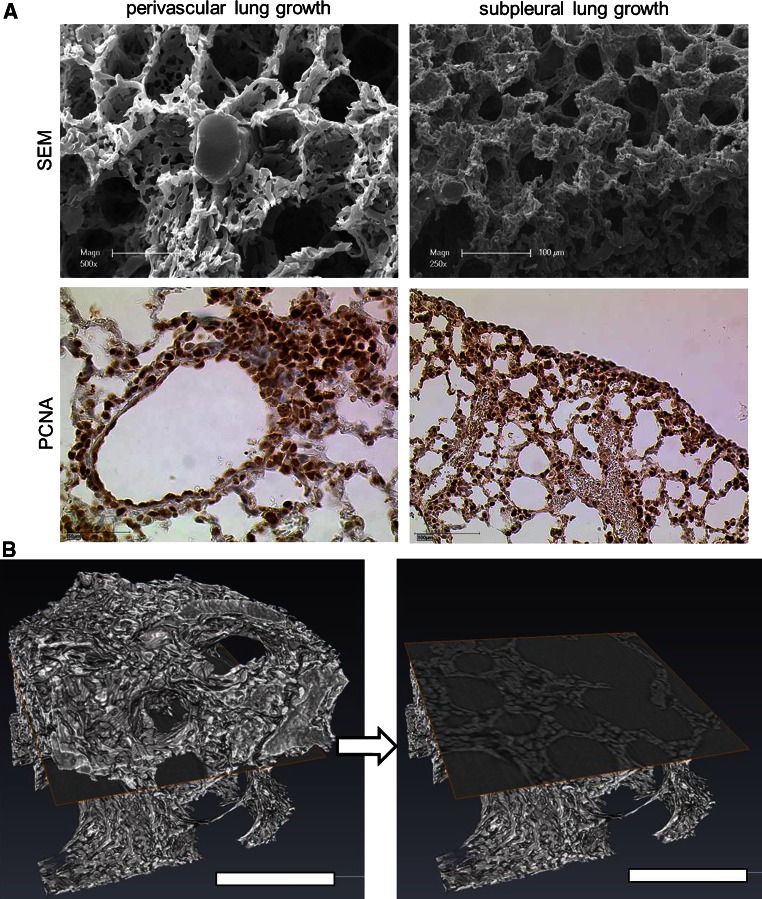
Spatial growth pattern of cardiac lobe. **a** The spatial heterogeneity of different lung growth can be seen in SEM-images of microvascular corrosion casts (*above*) and anti-PCNA-staining (*below*): perivascular lung growth located mainly in central parts of the lung (*left panel*) and subpleural lung growth (*right panel*) containing numerous anastomoses with pleural vessels. **b** High resolution synchrotron radiation tomography revealed the compact zones of vascular growth upon cross-section. *Bar* = 100 μm

A comparative evaluation of microvascular corrosion casts with anti-PCNA stained lung slides illustrates the spatial coherence of regenerative capillary growth after pneumonectomy (Fig. 2). Thus, a concentric perivascular growth pattern is evident, mostly in central parts of the cardiac lobe, whereas in peripheral zones a dynamic growth pattern can be observed in subpleural areas of the cardiac lobe. Hereby, a large number of anastomoses interconnect the pleural plexus with marginal vessels. As shown in Fig. 1d the subpleural growth pattern is decisively pronounced in regions of the cardiac lobe.

### Sprouting and intussusceptive angiogenesis

**Fig. 3:**
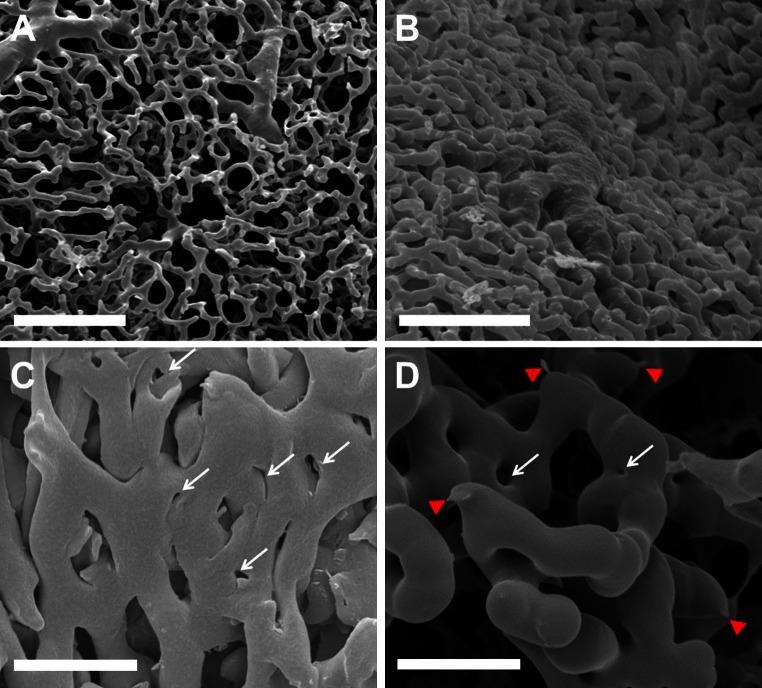
Sprouting and intussusceptive angiogenesis on pleural plexus. The pleural surface of the cardiac lobe is underlaid by a highly ramified capillary plexus originating from and draining to pulmonary vessel branches. **a** Coverage of approximately 15–25 % of the pleural surface. *Bar* = 50 μm. **b** 7 days after pneumonectomy this plexus increases in density, focally resulting in coverage of up to 85 %. *Bar* = 50 μm. **c** Numerous small caliber holes with diameters of between 1 and 5 μm as hallmarks of pillar formation (*white arrows*) show the high intussusceptive angiogenesis activity. *Bar* = 25 μm. **d** In less densely packed areas sprouts (*red arrowheads*) as well as pillars are evident. *Bar* = 20 μm

**Fig. 4:**
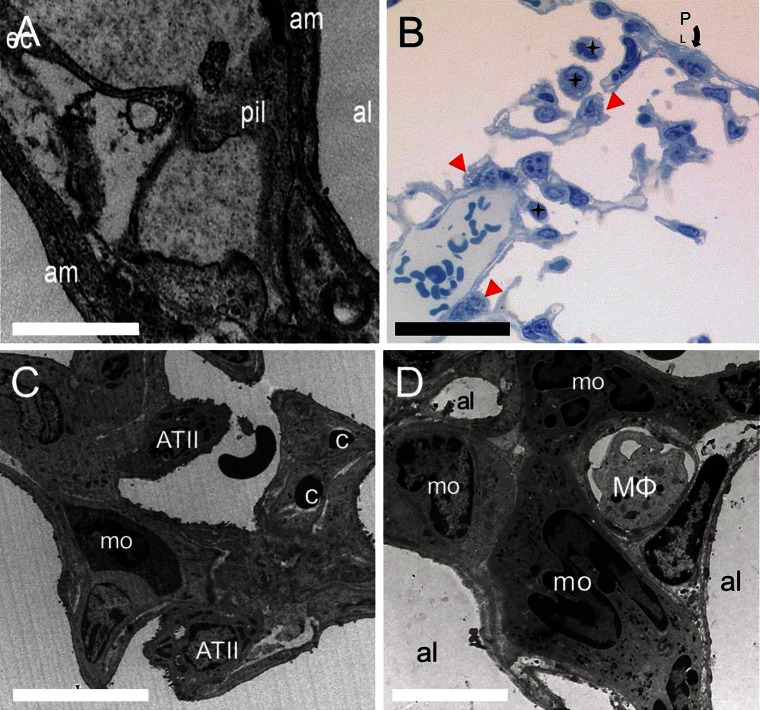
Transmission electron micrographs. **a** Transcapillary pillar formation indicates the occurrence of intussusceptive angiogenesis during compensatory lung growth which can be seen as protrusive interendothelial junctions. *Pil* denotes pillar, *al* alveolus, *am* alveolar membrane, *ec* endothelial cell; *Bar* = 10 μm **b** Semi-thin sections reveal the close spatial relationship between alveolar macrophages (*black stars*) and type II pneumocytes (*red arrowheads*), indicated in the subpleural region (*P*_*L*_ *: pleura*). *Bar* = 50 μm **c**, **d** Appearance of monocytes, alveolar macrophages, and type II pneumocytes indicates their proangiogenic relevance for alveolar morphogenesis. *ATII*, type II pneumocytes; *mo* monocyte; *c* capillary; *MΦ*; alveolar macrophage. **c** *Bar* = 15 μm; **d** *Bar* = 30 μm

The spatially heterogeneous compensatory lung growth is mainly driven by two different forms of angiogenesis: sprouting and non-sprouting (intussusceptive) angiogenesis. In the subpleural vascular plexus both forms are involved (Fig. 3). High resolution synchrotron radiation tomography revealed in growth zones around the major vessels predominantly intussusceptive pillars (Fig. 6c). The expansion of the pleural vascular plexus is carried out principally by the vasodilation and the incidence of intussusceptive pillars. In addition to our SEM-results, this finding was confirmed by the occurrence of transcapillary pillar formation in transelectron microsopy (Fig. 4a). The rapid expansion of central vascular network is paralleled by an extensive recruitment of pro-angiogenic cells as alveolar macrophages and pneumocytes type II. A subcellular analysis of alveolar cell structure revealed a close spatial relationship between pneumocytes type II and alveolar macrophages participating in alveolar morphogenesis of post-pneumonectomy lung growth (Fig. 4b–d).

### Formation of a new alveolar septum

**Fig. 5:**
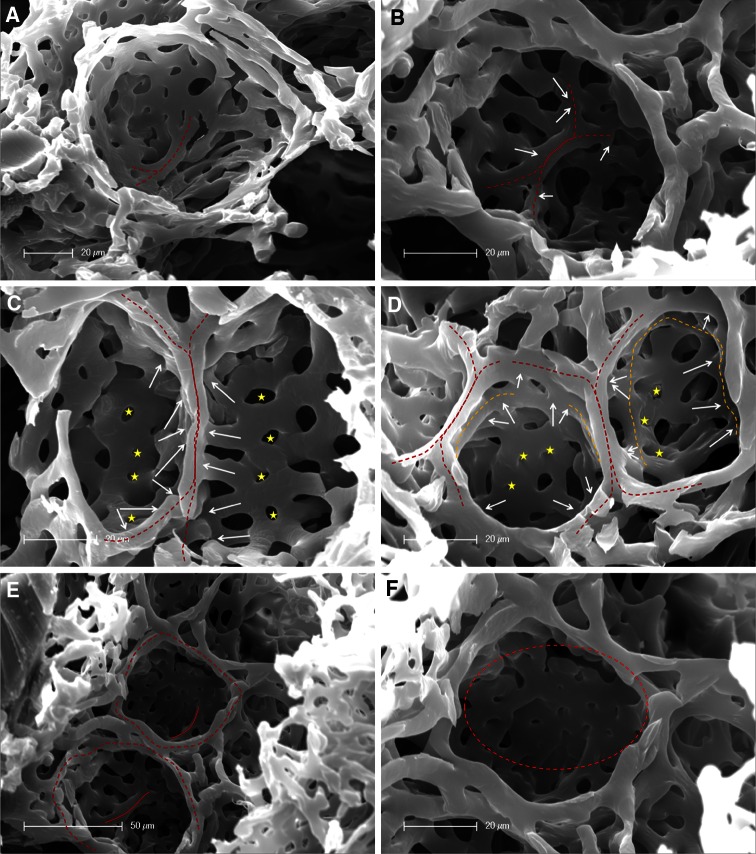
Corrosion casts of new alveolar septum formation. **a** In the midline of the alveolar cavity, an elevated vessel (*dotted red line*) starts to upfold, whereby another double-layered vessel crosses under it. **b** The septal vessel continues to elevate tightened dumbbell-shaped by two vessel at the end (*white arrows*). **c** A septal ridge (*red dotted line*) can be seen in the middle of the alveolar cavity whose local influxes (*white arrows*) are increasing by pillar formation (*yellow stars*) and vascular duplications. **d** Upfolding of a new alveolar septum (*red dotted line*) starts towards the alveolar entrance ring (AER). A second double-layered ring (*orange dotted line*) is developing by intussusceptive angiogenesis (*yellow stars*). **e** In the developed new alveoli with AERs (*red dotted lines*), an elevation and formation of new septa (*red lines*) is present in the midline. **f** Vascular remodeling modifies and expands the alveolar basket by the occurrence of pillars, typical hallmarks of intussusceptive angiogenesis (*circle*)

**Fig. 6:**
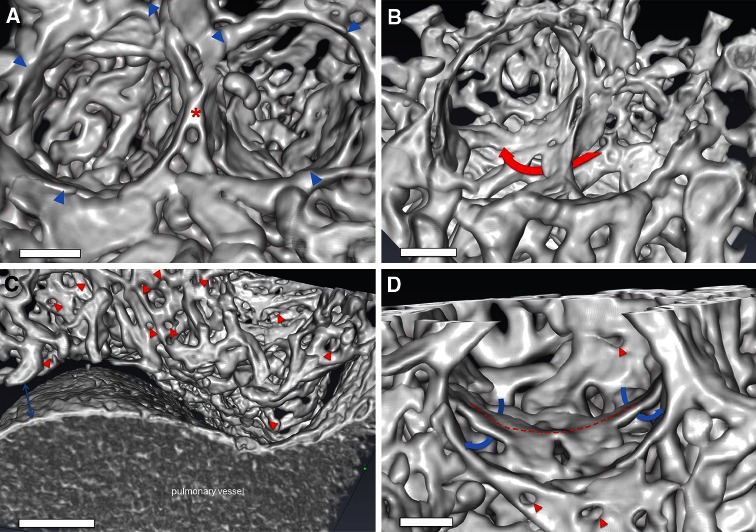
Synchrotron radiation tomographic microscopy. **a** Three-dimensional evaluation of microvascular corrosion casts by SRXTM illustrating the alveolar basket structure accompanied by the limiting AER vessels (*blue arrowheads*) and the elevated ridge (*asterisk*). *Bar* = 15 μm. **b** A typical example of double-layered vessels (*red arrow*) during alveolarization. *Bar* = 20 μm. **c** Analysis of central areas of the cardiac lobe revealed an increased density of intussusceptive holes (*red arrowheads*) indicative of the occurrence of intussusceptive angiogenesis around larger vessel structures. *Bar* = 60 μm **d** In lung alveolarization a ridge (*dashed line*) can be seen in the midline of the alveolar basket accompanied by double capillary layers (*blue arrows*) enabling the lifting-off of the inter-airspace septum. The rapid expansion by intussusceptive angiogenesis (*red arrowheads*) allows the pacing of isotropic lung growth after pneumonectomy. *Bar* = 10 μm (*see movies in supplemental material*)

The assessment of microvascular corrosion casts detected the evidence of new alveolar septum formation by alveolar duplication and capillary remodeling in compensatory lung growth after pneumonectomy. SEM- and high resolution SRXTM images of microvascular casts revealed in early stages of new alveolus formation an elevated vessel in the midline of alveolar cavity crossed by an underlaying vessel (Fig. 5a). The maturation of these alveolar septa continues by lifting off of this ridge-like vessel that is dumbbell-shaped connected to the perialveolar plexus vessels on both sides (Fig. 5b). The elevation of the ridge is accompagnied by the occurrence of intussusceptive angiogenesis ensuring a rapid expansion of the alveolar microvascular network and vascular duplications of the septal vessels (Fig. 5c). A second double-layered ring can be observed at the point when upfolding of the new alveolar septum reaches the same level as the alveolar entrance ring (AER) (Fig. 5d). In the completely developed alveoli, an elevated formation of new septa is evident in the midline (Fig. 5e). Maturation of alveolar vascular network is then modified and finished by the appearance of vascular remodeling and intussusceptive angiogenesis (Fig. 5f). A tomographic analysis of microvascular corrosion casts acquired by synchtron-radiation confirmed these observations (Fig. 6).

### Vascular remodeling of the alveolar entrance ring

**Fig. 7:**
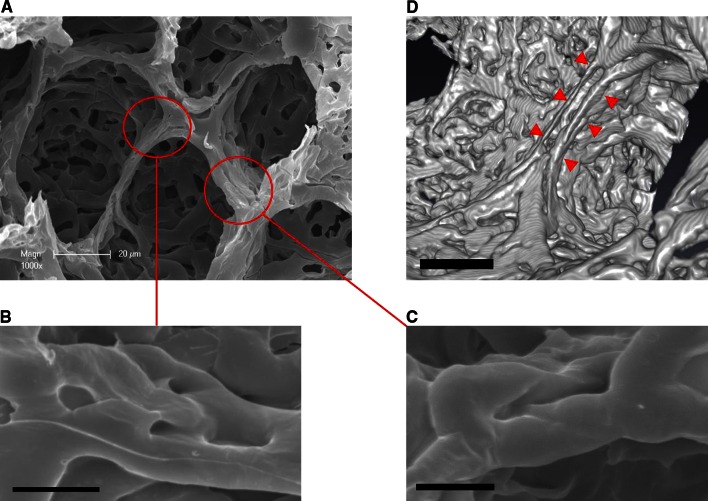
Vascular remodeling of the alveolar vascular ring. **a** Microvascular corrosion casts and SEM of vascular rings at the alveolar opening depict the incidence of remodeling due to intussusceptive angiogenesis. **b** The appearance of transluminal pillars on the interconnections between the alveolar entry rings indicates the occurrence of intussusceptive angiogenesis at the site of the alveolar opening. *Bar* = 15 um. **c** Elongation of such transluminal pillars results in a vessel duplication of ring blood vessels. *Bar* = 15 um. **d** High-resolution Synchrotron radiation tomographic microscopy demonstrates a vascular duplication (*red arrowheads*). *Bar* = 20 μm

Analysis of microvascular corrosion casts revealed a cylindrical morphology of alveolar ring vessels that are connected to the second tier vessels. Extensive structural changes in vascular morphology at the site of the alveolar entrance ring were evident. The effects of vascular remodeling could be observed observed in the appearance of intussusceptive angiogenesis at the site of the alveolar ring vessels (Fig. 7a). Transluminal pillars on the ring interconnections and vessel duplications on the ring boundaries (Fig. 7b, c) may emerge as a consequence of stretch due to ventilation pressure.

## Discussion

**Fig. 8:**
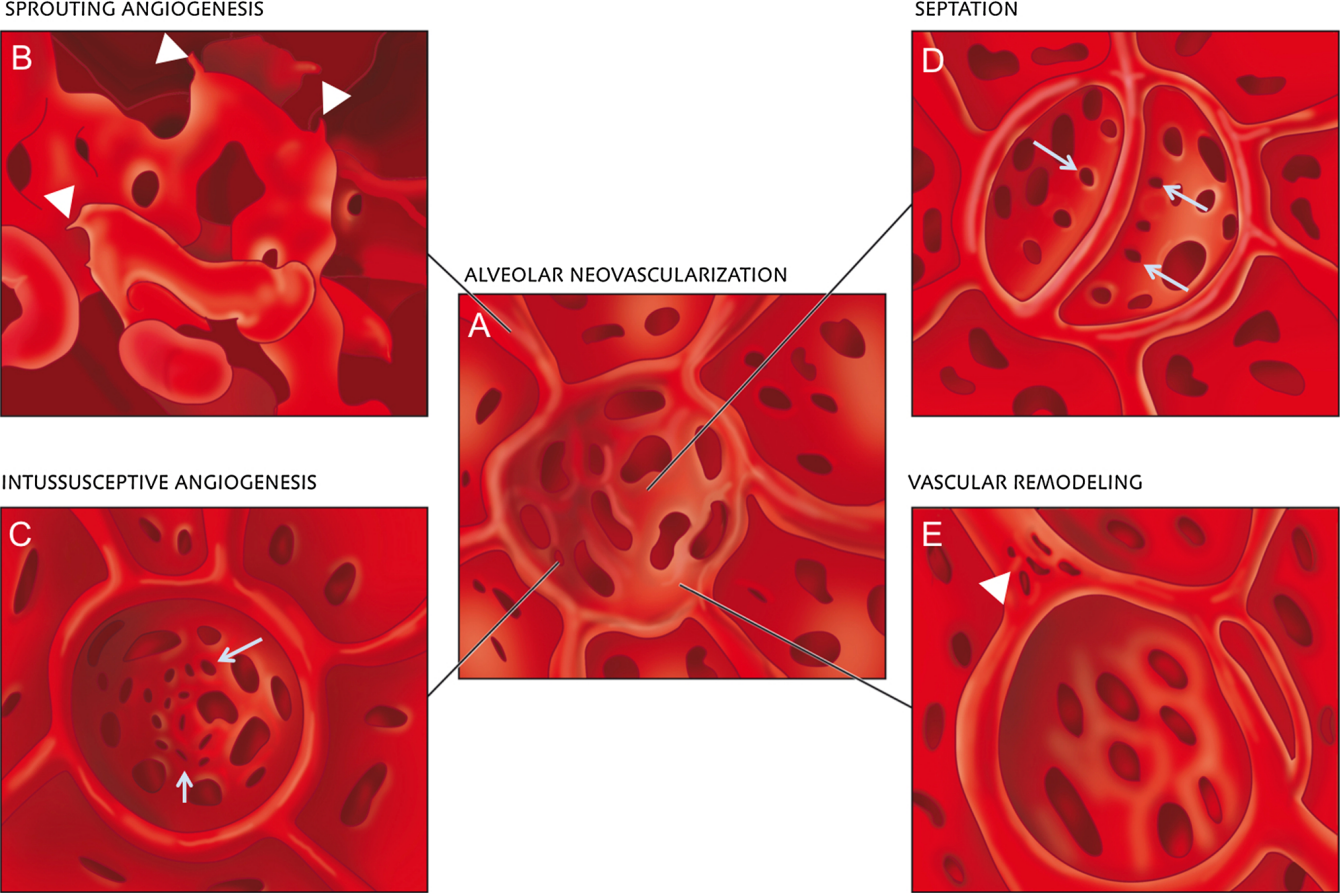
Schematic illustration of alveolar neovascularization (**a**) as seen in microvascular corrosion cast replicas with sprouting angiogenesis (**b**), which is frequently seen subpleurally and in compact growth zones. Sprouts are evident as blind ends or protrusions (*arrowheads*). **c** Alveolar intussusceptive angiogenesis, recognizable by the presence of numerous small caliber holes with diameters between 1 and 5 μm as hallmarks of pillar formation (*arrows*).** d** The formation of new alveolar septa is accompanied by the occurrence of parallely orientated intussusceptive pillars (*arrows*)—visisble in the cast as holes—ensuring a rapid expansion of the alveolar microvascular network. **e** Vascular remodeling also occurs on the AER vessels (*arrowhead*). Intussusceptive pillars may merge and split up the primary vessel

The present study looked at the morphogenetic evidence of angiogenesis in compensatory lung growth after pneumonectomy. The data obtained demonstrate the implication of sprouting and intussusceptive angiogenesis for alveolar tissue neoalveolarization on a sub-micron-scale using TEM, SEM and SRXTM, which is notably evident in subpleural capillary plexuses emerging from large caliber vessels. Additionally, there was a marked increase in the number of type II pneumocytes and alveolar macrophages in compensatory post-pneumonectomy lung growth. Furthermore, the findings indicate that neoalveolarization is closely associated with the upfolding of new alveolar septum formation and the appearance of features of lung development. Finally, analysis of corrosion casts revealed the occurrence of vascular remodeling in the form of intussusceptive angiogenesis at the site of the alveolar entrance ring (Fig. 8a–e).

The findings suggest the spatial dependency of lung growth after pneumonectomy can be observed constantly within the cardiac lobe. However, the indentation and cavity adjacent to the heart showed the highest proliferative activity. These findings may reflect the impact of a constant high tension due to cardiac movements on vascular growth. Another observation reflecting the evidence of angiogenesis as the main force in compensatory neoalveolarization was the expansion of the pleural plexus through sprouting und intussusceptive angiogenesis. The necessary supply of pleural and subpleural vessels is ensured, notably by the abundant supply of both the systemic bronchial and pulmonary vessels [13]. This pre-existing vascular pleural plexus might be assumed as being a prerequisite condition for alveolar construction. Hereby, intussusceptive angiogenesis might primarily represent a general fast recovery adaption to growth requirements [14] and to a rapid expansion of pre-existing pleural expansion.

Growth and elongation of intussusceptive pillars along the vessel axis between two branching points enable vessel duplications. In flatly extended vessel plexuses such as the perialveolar plexus, the intussusceptive pillars enlarge the meshwork width and vessel density for optimized gas exchange. Increases in alveolar size following the upfolding of the alveolar septa require increases in vessel coverage: a newly formed alveolus with a small diameter, e.g., 20 μm, requires significantly less vessels than are needed to cover a mature alveolus with a diameter of 70 μm.

In addition, our finding of a highly predominant occurrence of intussusceptive angiogenesis corresponds with the spatial heterogeneity of post-pneumonectomy lung growth which points to the importance and capacity of intussusceptive angiogenesis in tissue regeneration. This finding is consistent with our observation of high compaction of pillars abutted concentrically around the larger vessels in the cardiac lobe. Mechanical stress and the related changes in blood flow are thought to play pivotal roles in the initiation of the intussusceptive microvascular growth, in microvascular maturation and in remodeling. In mature alveoli, the appearance of pillar formation and vascular duplications at the site of the alveolar ring entrance could be seen. These scaffolding vessels contribute to the alveolar opening and bear mechanical stress.

In a similar manner, Wagner et al. [15] described an intense tissue remodeling in the visceral pleura after left pulmonary artery ligation as evidenced by a markedly increased amount of alveolar macrophages and type II pneumocytes. The transmission electron micrographs obtained also indicated that there was a marked increase in the number of macrophages, interstitial monocytes and type II pneumocytes in the alveolar airspace, after pneumonectomy. These findings are in line with our own previous flow cytometry data [16] on alveolar macrophage dynamics which demonstrated a significant upregulation of angiogenesis-related gene transcription in alveolar macrophages. In addition, the steep increase in the number of alveolar macrophages after pneumonectomy was related to local proliferation and not to blood-borne precursor cells. Pneumocyte type II cells participate in a way that is similar to that of the contribution of alveolar macrophages to alveolar angiogenesis. Recent studies on alveolar type II cell transplantation in rats have shown a stimulation of lung regeneration in the remnant lung after pneumonectomy [17].

As already shown in developmental alveolarization by Schittny et al. [18], we revealed a replication of existing alveoli by the upfolding of newly forming alveolar septa and capillary duplications. The microvascular growth and maturation of the alveolar capillary network is accompanied by the appearance of intussusceptive angiogenesis, which emphasizes the importance of intussusceptive angiogenesis in fast-expanding tissue regeneration. Double-layered vessels cover the bottom of the new alveoli and enable the lifting off of the new septa. Hence, the formation of a double-layered vascular network on the bottom of the alveoli due to intussusceptive angiogenesis is an incessant prerequisite for septal alveolarization after pneumonectomy, just as it is in lung development [19]. Recent clinical evidence suggests that alveolarization continues throughout childhood and adolescence in humans, thus underlining its important clinical implications for lung recovery in early human life [20].

In summary, this study provides evidence suggesting that compensatory lung regrowth is mainly driven by sprouting and intussusceptive angiogenesis. Our findings on structural neoalveolarization suggest that many mechanisms of developmental alveolarization might also be transferrable to post-pneumonectomy lung growth. These insights have a pronounced clinical significance for patients as far as the promotion of alveolar perfusion and lung capacity are concerned. On the basis of the results reported in this study, further molecular and functional studies are warranted for a substantiation of the observations presented here.

## Electronic supplementary material

Below is the link to the electronic supplementary material.

Supplementary material 1 (MPG 40574 kb)

Supplementary material 2 (MPG 45046 kb)
